# Exploring the length scales, timescales and chemistry of challenging materials (Part 2)

**DOI:** 10.1098/rsta.2023.0175

**Published:** 2023-10-30

**Authors:** Martin C. Wilding, Andrea Sella, Christopher A. Howard, Ana Jorge Sobrido, C. R. A. Catlow

**Affiliations:** ^1^ UK Catalysis Hub, Research Complex at Harwell, Rutherford Appleton Laboratory, Oxfordshire OX11, 0QX, UK; ^2^ Department of Chemistry, University College London, London WC1E 6BT, UK; ^3^ Department of Physics and Astronomy, University College London, London WC1E 6BT, UK; ^4^ School of Engineering and Materials Science, Queen Mary University of London, London E1 4NS, UK; ^5^ Cardiff Catalysis Institute, School of Chemistry, Cardiff University, Cardiff CF10 3AT, UK

**Keywords:** hybrid materials, catalysis, biomechanics, super-hard materials, low-dimensional materials, liquid structure, imaging

## Abstract

This themed issue explores the different length and timescales that determine the physics and chemistry of a variety of key of materials, explored from the perspective of a wide range of disciplines, including physics, chemistry materials science, Earth science and biochemistry. The topics discussed include catalysis, chemistry under extreme conditions, energy materials, amorphous and liquid structure, hybrid organic materials and biological materials. The issue is in two parts, with this second set of contributions exploring hybrid organic materials, catalysis low-dimensional and graphitic materials, biological materials and naturally occurring, super-hard material as well as dynamic high pressure and new developments in imaging techniques pressure.

This article is part of the theme issue 'Exploring the length scales, timescales and chemistry of challenging materials (Part 2)'.

## 1. PREFACE

In the second volume of this themed issue, the physics and chemistry of novel materials are further explored using a variety of advanced experimental and computational methodologies that probe the length- and timescales that define their physical properties. Over recent decades, major new classes of materials have been developed with novel technological applications. A detailed understanding of the structures and structure-related properties of these materials is, however, needed if the full potential of these materials is to be realized. In this volume we present a series of contributions that include studies relevant to physical chemistry, catalysis, biochemistry dynamic high pressure, and liquid structure, covering hybrid organic materials, low-dimensional and graphitic materials, biological materials and naturally occurring, super-hard materials. As with the earlier volume, the origin of this issue was a meeting held in June 2022 that honoured the scientific contributions of Professor Paul F. McMillan and this volume again showcases some of the emerging science in fields that McMillan had influenced.

Hybrid materials, or inorganic-organic compounds are important and technically interesting materials since new properties and applications can emerge and their dimensions and compositions can be tuned to optimize particular properties The thermochemistry of these materials is complex and the first contribution to this second volume of the themed issue is a review of their thermochemical properties by Alex Navrotsky & Gerson Leonel [1]. The results of calorimetry measurements for three classes of hybrid materials are discussed. Metal organic frameworks (MOFs) comprise metal nodes linked by organic molecules to form three-dimensional, porous networks that have applications that can include catalysis, waste remediation, water purification and as pharmaceuticals [2,3]; and the porosity of MOFs, their surface areas and topology are often compared with zeolites. Calorimetry measurements discussed by Navrotsky & Leonel [1] show that the enthalpies of formation of MOFS follow a similar trend to those of zeolites and that their thermodynamic stability also decreases with increased pore volume. As with zeolites, the energetics of MOFs level off at high porosity and suggest that the interiors of the larger pores have little effect on their stability. Within MOFs an important subclass of materials is the zeolitic imidazolate frameworks (ZIFs) [4], which have also been studied by Navrotsky and Leonel [1]. These calorimetry measurements identify the increased stability of phases produced by increased grinding mechanochemical synthesis.

Polymer derived ceramics (PDCs) are hybrid materials with many potential applications, which include ceramics containing silicon directly coordinated by carbon and are produced by pyrolysis of silicon containing polymers resulting in systems that can contain SiCN, SiO and SiOC not achieved by conventional sintering [5]. Several PDCs have been studied by Navrotsky's group to evaluate the most favourable energetic interactions which include mixed bonding effects, important in stabilizing amorphous forms of PDC. A conceptually simpler class of hybrid materials are those in which large organic cations replace inorganic ions in a conventional structure, including those formed with formate anions connecting divalent metal cations which are also are reviewed in Navrotsky and Leonel's contribution to this volume and which show significant thermodynamic stability with respect to binary oxides and formic acid, indicating that these compounds are persistent and can have their compositions for different applications [6].

Zeolites remain one of the most important classes of porous materials and they are extensively used in commercially important catalysis applications [7–9]. The molecular diffusion within the zeolite pores is an important influence on the efficacy of catalytic reactions and neutron scattering techniques, with their sensitivity to hydrogen, can be important probes of the dynamics of the diffusing species. In the study of Matam *et al.* [10] Quasi Elastic Neutron Scattering (QENS) [11,12] is used to determine the diffusion of methanol in a series of zeolites at different temperatures is outlined. The zeolite catalyst studied, ZSM-5, is important in methanol to hydrocarbon (MTH) reactions and understanding the diffusion of methanol in these materials is accordingly both environmentally and commercially important since alternative methods of producing gasoline, olefins, and aromatics without relying on fossil fuels are key in achieving net zero carbon emission targets. QENS data have been acquired at the ISIS neutron source for different ZSM-5 zeolites which have different Si/Al ratios, and the results are compared with those from molecular dynamics simulations which probe similar timescales. The different Si/Al ratios determine the number of Brønsted acid sites which strongly influence methanol diffusion [11,12]. As the Si/Al ratio is increased, the fraction of mobile methanol increases and the nature of the methanol dynamics also changes from rotational to translational diffusion. Where the Brønsted acid site density is high, the diffusion of methanol is influenced both by adsorption onto the acid sites and intramolecular interactions that result in the formation of methanol clusters within the zeolite pores reducing overall mobility. This insight into the complex diffusion dynamics of methanol highlights the advantages of combining different experimental and computational techniques and how, by developing a detailed understanding of processes over different time and length scales, more efficient processes can be designed if important, sustainability goals such as net-zero carbon emissions are to be realized.

Catalysis is further explored in the paper by Ben-Jaber *et al.* [13]. In this study surface enhanced Raman spectroscopy [14] is used to evaluate photocatalytic processes, with photo-induced Raman Spectroscopy (PIERS) [15] employed to study the development of photogenerated oxygen vacancies in thin TiO_2_ films. Oxygen vacancies are generated first by reduction of Ti^4+^ to Ti^3+^ using ultraviolet radiation and dynamic Raman spectroscopy then monitors the defect kinetics using rhodamine-6G dye as a target molecule. A series of spectra collected over a period of time shows the progressive decrease in intensity following an initial enhancement which is attributed to the role that the oxygen defects play in the enhanced charge transfer. The chemical enhancements outlined in this study indicate that materials that can be engineered with deep oxygen vacancy sites may be highly effective photocatalysts and that PIERS has the potential to be an important tool in evaluating the efficiency of photocatalytic materials.

The papers collected in these two themed issues emphasize how understanding the length and timescales that control material process and properties cross disciplinary boundaries as illustrated effectively in the contribution by Filip Meersman and colleagues [16], who use high pressure techniques and X-ray diffraction to evaluate the mechanical properties of biomolecular assemblages. Amyloid fibrils have been studied extensively, primarily because of their association with neurodegenerative diseases [17], although it is now recognized that their occurrence is more widespread, and they are viewed as playing an essential part of polypeptide chain behaviour. The potential use of amyloid fibrils in bioengineering applications means that their mechanical strength is of particular interest and Meersman *et al.* [16] present diffraction patterns for microcrystalline samples encapsulated in a diamond anvil cell to pressures of 12.8 GPa; the structures become disordered above 8 GPa so Meersman *et al.* have used these data up to 1.4 GPa to obtain their bulk modulus. The microcrystals are tightly packed, and compression of these fibrils is anisotropic; the longer, crystallographic *a*-axis is the least compressible while the *c*-axis, corresponding to the inter-sheet distance, is more compressible suggesting that the mechanical behaviour of these biomaterials is related to both packing and hydrogen bonding.

High pressures and high temperatures, or extreme conditions, result in interesting physics and chemistry, as has been discussed in the first volume of this themed issue. High pressure can be both static, as outlined above, or dynamic. Dynamic high pressure that can result from shock or impact is discussed from two very different perspectives in two contributions to this volume. In the paper by Read *et al.* [18] the very real effects of high pressure are discussed in a study designed to evaluate the performance of fragmentation capture materials and in devising metrics for assessing the performance of personal protective equipment. The standard material from fragmentation capture is strawboard, which is, however, not always currently available. Read and colleagues have developed a series of experiments to compare strawboard with alternative materials, which include studying the behaviour in response to different impact velocities of particles of known geometry either fired from a gas gun or from explosive fragmentary configurations. Strawboard was compared with medium density fibreboard (MDF) and carpet underlay, and it has been shown that not only is MDF a more widely available and cheaper alternative to strawboard but is also a superior material when subject to single impart and explosive testing scenarios. Accordingly, this study shows that MDF has the potential to be adopted as an industry standard for fragmentation capture.

In the natural world, impacts from meteorites can result in the formation of dramatic landscape features, such as the famous Meteor Crater in Arizona. High temperatures and pressures can be generated by these impacts, however the formation of the meteorites themselves also involves high pressure and temperature and high pressure mineral phases can be recovered from meteorite samples. In the paper by Péter Németh *et al.* [19], diamond-like grains recovered from the Canyon Diabolo Meteorite (that formed Meteor Crater) have been studied using electron and X-ray diffraction techniques and high-resolution transmission electron microscopy (HRTEM). Collectively these hard carbon grains are referred to as Lonsdaleite since early X-ray diffraction patterns indicated that this hexagonal diamond phase was present [20]. However, subsequent diffraction studies have shown that the peaks are quite broad and do not represent a single phase and that the hard carbon grains may be formed form both cubic and hexagonal diamonds. Németh *et al.*'s further investigation into the structure of these grains suggests an even more complex structure to these intriguing materials: both electron and X-ray diffraction patterns are characteristic of materials with stacking disorder and there are reflections corresponding to the interlayer spacing of graphene. In addition, there are domains of ‘diaphite’ (graphite-diamond nanostructures) within the hard carbon grains as well as both graphitic and graphene regions linking different domains [21,22]. From their study Németh *et al*. conclude that the hard carbon grains found in the Canyon Diabolo Meteorite are complex nanocomposites comprising diamond with hexagonal and cubic stacking disorder intergrown with diaphite.

Carbon-based materials are of interest because of their many potential applications; diamond and the hard carbon grains discussed by Nemeth *et al.* [19] have obvious applications as super hard materials and since its isolation, graphene has been recognized as an important component of semiconducting electronic devices [23]. There is interest too in related graphitic materials including carbon nitrides, since these latter materials are easier to produce, have similar properties and their applications are more scalable. There are two contributions to this volume focusing on these graphitic carbon nitrides, especially on the spontaneous dissolution of these materials in aqueous and other polar solvents [24,25]. Polytriazine Imide (PTI) is a polymeric carbon nitride formed from triazine groups linked by NH bridges. The material is routinely synthesized using a molten salt route and forms large planar sheets with intercalating lithium halide retained from the synthesis [26]. The PTI dissolves into polar solvents spontaneously, without the need for mechanical agitation, meaning that the two-dimensional sheets remain intact and so this process offers a low energy route to solution processing of these carbon nitrides [27]. In the paper by Lisowska *et al*. [24] the chemistry of PTI solutions is explored as a function of pH, using aqueous solutions of common acids and bases. The solutions are studied using UV-Visible spectroscopy; there are characteristic absorption bands assigned to different parts of the PTI structure with spectra collected for both PTI intercalated with lithium halide and an intercalant-free PTI, in which the salt has been removed by Soxhlet extraction. The amount of PTI dissolved in the solutions is determined by the intensity of these peaks. The dissolution of PTI depends on the pH of the aqueous solution and has a maximum at a pH of 10.2; the PTI ‘salts out’ at higher pH. The acid-base reactivity in these solutions is further explored through DFT calculations. It is suggested that the PTI dissolves in the solutions as a negatively charged species with mildly acidic or basic pH providing sufficient net charge on the two-dimensional sheets to promote dissolution.

Nanoparticle dispersions such as the PTI solutions are often viewed as colloidal suspensions. However, in true colloidal suspensions the particles are large, while the size of the PTI nanosheets is typically only 100 nm in diameter and the sheets themselves are only a few layers thick (approx. 2.5 nm). The nanoparticles in these solutions therefore have dimensions that compare with the surrounding solvation shells and the influence of the solvent can provide the thermodynamic driver force for spontaneous dissolution, suggesting that nanoparticle solutions, such as the PTI solutions, are intermediate between a simple solution and a true colloidal suspension. The rearrangement of solvent molecules around PTI nanosheets is explored using X-ray and neutron scattering, combined with empirical potential structural refinement (EPSR) [25]. Solutions of PTI in the aprotic polar solvent dimethyl formamide (DMF) are investigated by both high energy X-ray diffraction and neutron diffraction with isotopic substitution; comparison of the X-ray data for the PTI solution and the DMF solvent shows obvious changes in liquid structure in both reciprocal and real space. These differences persist to a least 10 Å and indicate substantial reordering of the DMF solvent. Differences in the neutron patterns are more subtle, confirming that the structure are seen in C–C, C–H, C–O and C–N contributions rather than as changes in the distribution of hydrogen. The solvent ordering is modelled by using a single layer of the carbon nitride generated from the crystal structure; the c-axis is extended, and the space filled with MDF molecules and Br- ions. Since the PTI is intercalated with LiBr, the Br- ions enter the solution during exfoliation, while Li^+^ ions are retained in the PTI nanosheet resulting in a net positive charge. Both diffraction data sets are used to constrain this EPSR model. In the modelled solution the Br- ions are arranged close to the PTI surface, normal to the PTI surface clearly defined solvation shells are developed reflecting the interaction of the carbon nitrogen and oxygen in the DMF molecule with the charged PTI layer. These solvent ordering effects lower the free energy of the system and provide the energetic advantage that favours dissolution of PTI.

Wide- and small-angle X-ray scattering is combined with EPSR modelling in the study of perfluroalkyl substances (PFAS) presented by Benmore *et al.* [28]. Exposure to these chemicals can have an adverse impact on human health and contamination of water by PFAS is becoming increasingly more commonplace and severe [29]. These substances, particularly the short-chained variants, can persist in the aquatic environment and remediation is difficult. This study of concentrated PFAS solutions is one of the few recent studies of fluorinated liquids and is designed to enable a deeper understanding of the structure of these liquids that will assist in addressing this environmental challenge. Solutions of perfluoroalkyl carboxylic acids with different chain lengths were investigated; these molecules have a hydrophilic and hydrophobic part, and it has been suggested that the interaction of these molecules with water can result in nanoscales segregations. The small angle scattering data show changes in a peak position and intensity as a function of chain length and indicate the periodicity of a nanoscale heterogeneity. The presence of the perfluoralkyl carboxylic acids dramatically disrupts the water structure and the interactions over multiple length scales include the development of interactions between non-bonded clusters of perfluoralkyl molecules and the formation of solvation shells comprising dense chains of water molecules with distorted hydrogen bonds surrounding clusters of the acid molecules.

Throughout this volume the different contributions together illustrate how state-of-the-art experimental techniques can be used to explore the time- and length-scales that determine the chemical and physical properties of a range of different materials. It is therefore fitting that the final contribution to this themed issue is an overview of an advanced X-ray technique developed for high resolution chemical imaging. In the paper by Omori *et al.* [30], details of the X-ray scattering based computational tomography technique are outlined; they resemble the more familiar computational tomography (CT) technique that is based solely on the attenuation of X-rays in that it provides compositional information at high resolution and provides detailed three-dimensional reconstruction of complex materials. The X-ray scattering-based CT technique provides structural information in the form of wide-angle scattering data (X-ray diffraction and pair distribution function analysis) as well as small angle X-ray scattering data that provide structural information, in addition to the chemical information obtained from absorption contrast. In this review, Omori *et al.* [30] discuss the many different applications of this technique, including materials science, catalysis and biosciences as has been discussed elsewhere in this volume, further emphasizing the advantages of probing different length- and timescales in order to understand complex processes and properties.

We would like to thank all of the contributing authors for giving their time and energy to enable the publication of the collection of articles that comprise this volume and hope that these papers illustrate how the application of different combinations of experimental and computational techniques provide an opportunity to understand the processes and properties of complex materials relevant to a range of different disciplines; and that an appreciation of how these techniques are applied across this range can help develop more unified approaches to understanding structure and structure-related properties.

We are grateful to the Royal Society for providing us with the opportunity to develop this themed issue and would especially like to thank the commissioning editor Alice Power for her assistance in producing both volumes.

## Data Availability

This article has no additional data.
